# Sinonasal *DICER1*‑mutated embryonal-like (botryoid-like) rhabdomyosarcoma in an adult: report of the first case

**DOI:** 10.1007/s00428-025-04358-1

**Published:** 2025-12-03

**Authors:** Miguel Rito, Sofia Fernandes, Robert Stoehr, Abbas Agaimy

**Affiliations:** 1 Instituto Português de Oncologia de Lisboa, Francisco Gentil, Lisbon, Portugal; 2 Institute of Pathology, University Hospital Erlangen, Friedrich-Alexander University Erlangen-Nürnberg (FAU), Erlangen, Germany; 3 Instituto de Anatomia Patológica, Faculdade de Medicina da Universidade de Lisboa, Lisbon, Portugal

**Keywords:** Molecular profiling, Cancer predisposition syndrome, Head and neck, Germline mutation, Nasal chondromesenchymal hamartoma

## Abstract

The *DICER1* gene, essential for microRNA biogenesis and posttranscriptional gene regulation, has been implicated in a variety of benign and malignant neoplasms, particularly within the context of the *DICER1*-related tumor predisposition syndrome. While *DICER1*-associated rhabdomyosarcomas (RMS) are predominantly documented in the genitourinary tract, we present the first case of a *DICER1*-mutated embryonal-like (botryoid-like) RMS of the nasal fossa. A 63-year-old woman without relevant family history presented with nasal obstruction, headaches, and epistaxis and underwent resection of a polypoid sinonasal mass. Histopathological analysis revealed a spindle cell neoplasm with prominent botryoid growth, rhabdomyogenic features, and foci of metaplastic cartilage. Immunohistochemistry demonstrated positivity for desmin, myogenin, and MyoD1, prompting molecular testing that confirmed pathogenic *DICER1* and *KRAS* mutations. Germline testing was negative for *DICER1* alterations, and the *DICER1* variant was determined to be somatic. The covering respiratory epithelium showed prominent hyperplastic changes, in areas closely mimicking biphenotypic sinonasal sarcoma. Targeted RNA sequencing revealed no gene fusions involving *MAML3*, *FOXO1*, *PAX3*, or other genes. This case underscores the broad differential diagnosis of spindle cell lesions of the sinonasal tract and highlights the utility of combined morphology, immunohistochemistry, and molecular testing in establishing a diagnosis. Notably, the presence of cartilage foci within a RMS-like neoplasm represents a strong clue to an underlining *DICER1* alteration. The rarity of this presentation in the nasal fossa at this age, coupled with its implications for diagnosis, treatment, and familial screening, emphasizes the need for awareness of the morphology patterns of *DICER1*-associated neoplasms across diverse anatomical sites.

## Introduction

The *DICER1* gene is located on chromosome 14q32.13 and encodes a protein that is a ribonuclease (RNase) III endoribonuclease and a key component of the RNA interference pathway. Through regulation of microRNA (miRNA) biogenesis, *DICER1* regulates gene expression on a posttranscriptional level, and, hence, influences tumorigenesis [1].

Disease‐associated variants in *DICER1* were first described in 2009, associated with familial pleuropulmonary blastoma [1]. Since then, several benign and malignant entities have been added to the list of *DICER1* associated neoplasms, including pediatric cystic nephroma, embryonal rhabdomyosarcoma of the uterine cervix, ovarian Sertoli‐Leydig cell tumor, nasal chondromesenchymal hamartoma, ciliary body medulloepithelioma, multinodular goiter, differentiated thyroid carcinoma, pituitary blastoma, and unusual sarcomas of various sites, including the uterine cervix, kidney, brain, and peritoneum, amongst others.

Most patients with *DICER1*-associated neoplasms have a predisposing germline, loss‐of‐function *DICER1* variant, causing the so-called *DICER1*-related tumor predisposition syndrome [1]. Inherited in an autosomal dominant manner, this syndrome usually has its first manifestations in children and young adults. A small subset of patients has a single sporadic tumor harboring acquired tumor-confined biallelic *DICER1* pathogenic variants, and therefore, they lack other manifestations of the syndrome [1].

Rhabdomyosarcoma (RMS; embryonal type) is the most common sinonasal sarcoma in children, mostly arising before the age of 5, although patients of all ages may rarely be affected [2]. Embryonal RMS (ERMS) displays primitive mesenchymal cells in various stages of myogenesis, within a loose, myxoid mesenchyme, with alternating areas of cellularity. Grossly, ERMS may present either as a poorly circumscribed, fleshy, pale-tan mass that invades through neighboring structures, or as polypoid submucosal tumors, protruding within mucosal/epithelial-lined cavities with a characteristic botryoid pattern. The latter has been described mostly in the genitourinary tract but can occur in any other cavity-bearing organ, albeit distinctly rare [3].

We herein describe the first case of a *DICER1*-mutated sporadic botryoid-like (embryonal-like) RMS of the nasal fossa of a 63-year-old woman.

## Clinical history

A 63-year-old woman presented with a 4-month history of nasal obstruction, headaches, and epistaxis. Otolaryngology examination and MRI identified a mass occupying the left nasal fossa from the middle meatus to the floor. A preliminary biopsy from an outside institution suggested “sinonasal glomangiopericytoma.” Endoscopic surgery was performed, and the lesion was resected in piecemeal.

Grossly, the surgical specimen was composed of multiple irregular, white-yellowish fragments with hemorrhagic areas, partially covered by mucosa, with dimensions ranging between 4 cm and 0.5 cm in their largest axes. The larger fragments had a polypoid configuration.

**Fig. 1:**
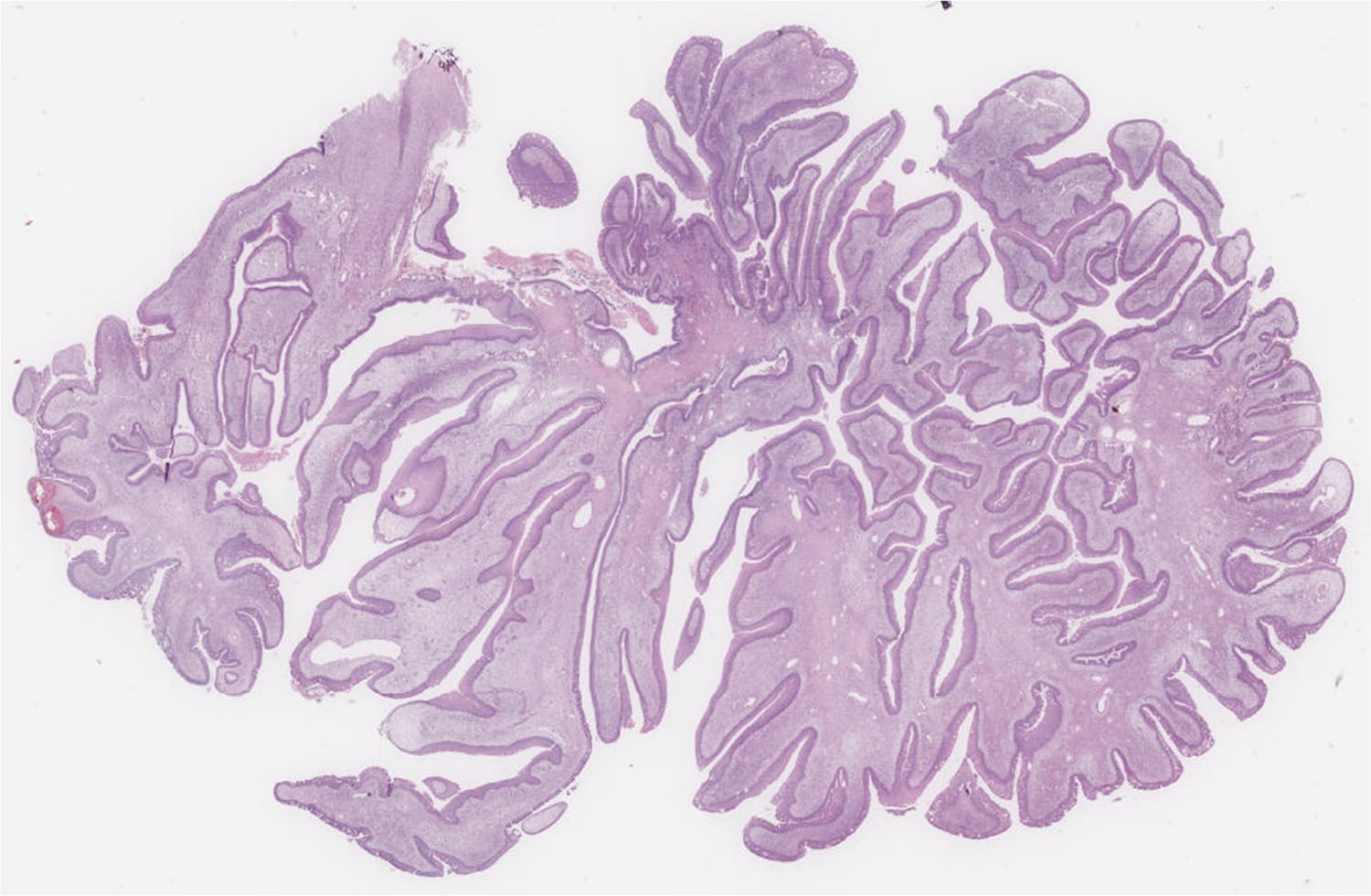
Low power whole-mount view of the lesion showing exophytic, polypoid/papillary (botryoid) configuration.

**Fig. 2:**
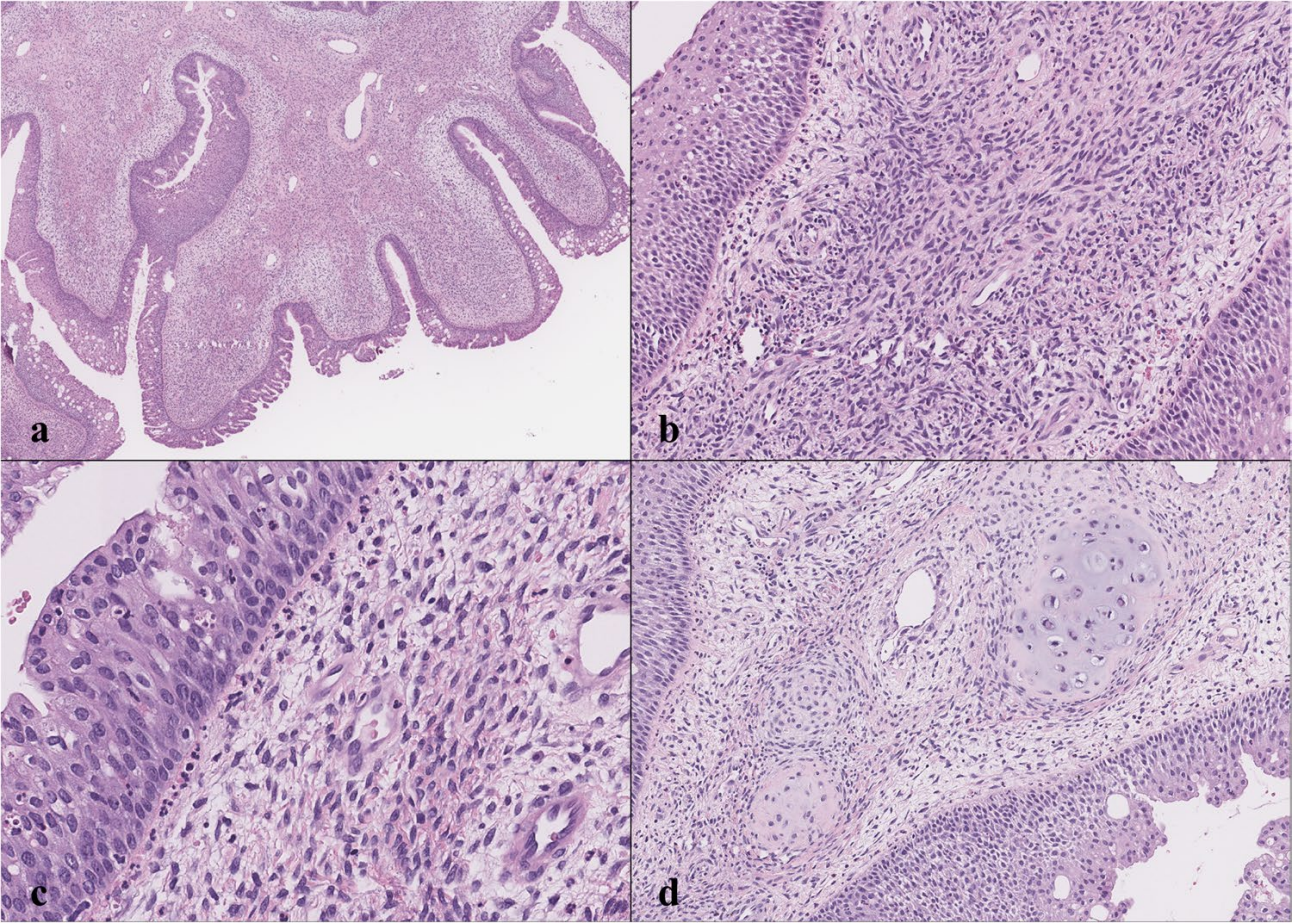
The papillae were covered by respiratory, transitional metaplastic, and squamous epithelium, with hyperplastic/papillary reactive changes and focal invaginations reminiscent of biphenotypic sinonasal sarcoma (**a**). Submucosal hypercellular relatively monotonous spindle cell proliferation, with a diffuse and fascicular pattern, set in a variably loose, myxoid, stroma was seen (**b**, **c;** note metaplastic squamous epithelium on top in **b**). Well-defined foci of cartilage were embedded within the spindle cell tumor (**d**).

Histologically, multiple fragments with polypoid/papillary configuration were seen (Figs. 1 and 2), covered by respiratory, transitional metaplastic, and squamous epithelium, extensively infiltrated by inflammatory cells and with prominent hyperplastic (papilloma-like) reactive changes. The submucosal stroma was occupied and expanded by a monotonous spindle cell proliferation with a diffuse and fascicular pattern, set within a variably fibrous to occasionally loose, myxoid stroma. There were no marked cytological atypia or necrosis, but mitotic activity was evident with > 10 mitoses/10 HPF. Multiple well-defined foci of mature-looking cartilage were scattered within the stroma. The overall appearance was very suggestive of botryoid-type ERMS, but with variable resemblance to biphenotypic sinonasal sarcoma in areas with invaginated hyperplastic respiratory epithelium. A cambium layer was not seen beneath the surface epithelium.

**Fig. 3:**
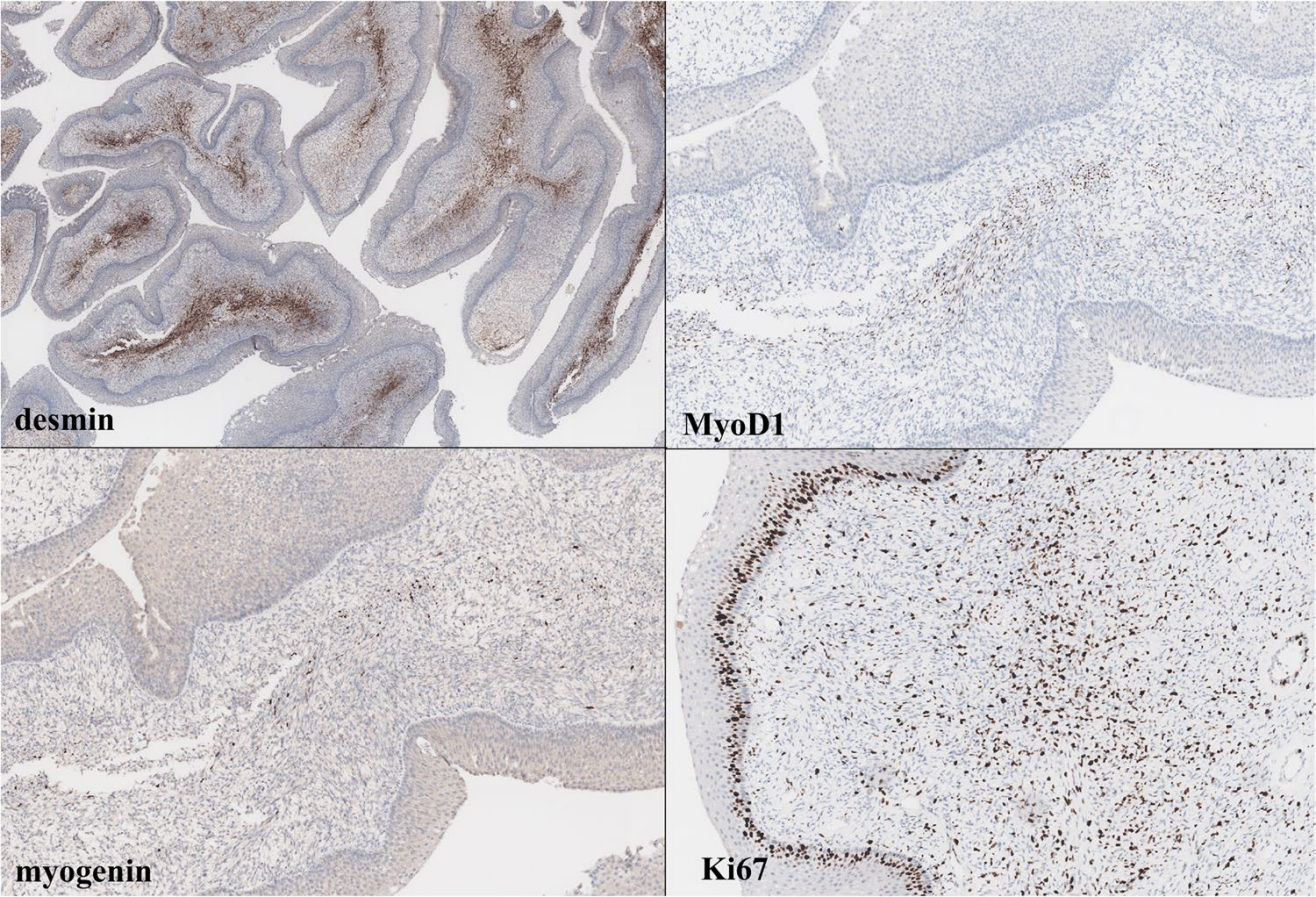
Staining with desmin, myogenin, and MyoD1 was observed in the central cores of the botryoid-like leaflets, more evident with desmin. Ki67 proliferative index approaches approximately 25% in the “hotspots”.

Focal staining with desmin, myogenin, and MyoD1 was observed in the central cores of the botryoid-like leaflets (Fig. 3). Apart from positivity with AE1/3, CAM5.2, and EMA in the surface epithelium, and S100 in the cartilage foci, all other remaining markers were negative in the tumor cells (S100, SOX10, beta-catenin, EMA, smooth muscle actin, CD34, LEF1, STAT6, panTRK, and preserved H3ME). The proliferative index (Ki67) was approximately 25% in “hotspots.”

The overall botryoid-like appearance, combined with variable rhabdomyogenic features and cartilaginous foci, leads to the suspicion of a *DICER1*-associated malignancy. Molecular testing on isolated tumor DNA (using the Illumina TSO500 DNA Panel) revealed a pathogenic *DICER1* mutation (c.5437G>C, p.Glu1813Gln, Variant Allele Frequency (VAF): 65%), along with a pathogenic *KRAS* mutation (c.35G>T, p.G12V, VAF: 11%). Targeted RNA sequencing (TruSight RNA Panel, Illumina) revealed no gene fusions.

These molecular results, along with the tumor morphology and immunophenotype, are consistent with a *DICER1*-associated embryonal-like (botryoid-like) RMS with foci of metaplastic cartilage.

The patient was further treated with local radiation therapy after surgery and remains free of disease with a 6-month follow-up. Exploration of the patient’s personal clinical history was negative for *DICER1*-related neoplasms. Her family history was negative as well for *DICER1*-related neoplasms (sister with “colon cancer” at age 56 years and grandmother with “uterine cancer” at age 64 years).

Due to the fact that *DICER1* alterations are frequently an indication of a hereditary disease, a germline molecular study of the *DICER1* gene was performed after informed consent. The DNA from peripheral blood was analyzed by NGS (SureSelectXT HS, Agilent), and no pathogenic single-nucleotide or copy number variants were identified.

## Discussion

In this report, we describe a *DICER1*-associated embryonal-like (botryoid-like) RMS with foci of cartilage originating in the nasal fossa of an adult female patient. To the best of our knowledge, this is the first documentation of this kind of neoplasm at this location.

From a diagnostic point of view, it is important to mention that this case represents a monomorphic spindle cell lesion of the sinonasal tract, whose differential diagnosis is wide and includes several entities like biphenotypic sinonasal sarcoma, glomangiopericytoma, neural (schwannoma, malignant peripheral nerve sheath tumor) or muscle tumors (leiomyoma, leiomyosarcoma, or spindle cell RMS), angiofibroma, solitary fibrous tumor, or even spindle cell carcinoma or melanoma. To reach a diagnosis, apart from morphology, usually a selected immunohistochemical panel, along with molecular testing, is needed to reach a final diagnosis [2, 4]. In our case, the overall botryoid-like appearance, combined with variable rhabdomyogenic features, highlighted by the positive immunohistochemical staining with desmin, myogenin, and MyoD1, along with the presence of cartilaginous foci, leads to the suspicion of a *DICER1*-associated malignancy, later confirmed by molecular testing.

ERMS is associated with several syndromes, including *DICER1*-related tumor predisposition syndrome*.* Nevertheless, in this latter setting, the affected sites are essentially the genitourinary (GU) tract, namely the cervix or bladder. Other GU tract sarcomas have also been reported (undifferentiated sarcoma of the ovary, ovarian fibrosarcoma, adenosarcoma of the female genital tract, peripheral primitive neuroectodermal tumor of the uterine cervix, and anaplastic sarcoma of the kidney) and sarcomas outside this location too (soft tissue leiomyosarcoma, intracranial spindle cell sarcomas, and pleuropulmonary blastoma-like peritoneal sarcomas). However, sarcomas in the *DICER1* setting are generally very rare [3, 5, 6].

Despite its rarity, it became evident that *DICER1*-associated sarcomas mostly share morphological features irrespective of their site of origin. These characteristic features include undifferentiated small round blue cells, spindle cells, foci of anaplasia (large, bizarre pleomorphic cells), rhabdomyoblastic differentiation (with expression of skeletal muscle markers myogenin and myoD1), chondroid differentiation, and bone/osteoid formation (suggesting origin from pluripotent cells). Although not all of these features are present in every case, identification of this unique morphological appearance should raise suspicion of a *DICER1* association, and tumor testing should be considered [6]. This was our approach when these features were identified. These shared morphological features are observed irrespective of the *DICER1* alteration being germline or somatic.

Moreover, *DICER1*-altered ERMS formed a distinct cluster using unsupervised hierarchical clustering epigenetic studies that clearly segregated from the other clusters of *DICER1*-wt ERMS, alveolar RMS, and *MYOD1*-mut RMS [7]. Additionally, from a clinical point of view, a recent study by Antonescu et al. [8] showed a better recurrence-free and disease-specific survival for *DICER1*-mut ERMS compared to *DICER1*-wt cases, further justifying this segregation.

Apart from nasal chondromesenchymal hamartomas as a genuine component of the *DICER1*-related tumor predisposition syndrome, *DICER1*-altered head and neck lesions are strictly rare. Our case is the first documentation of a *DICER1*-mut RMS in an older adult. Our literature search uncovered one *DICER1*-mutated ERMS of the maxilla of a 6-year-old boy, which was retrospectively identified in the DNA methylation study previously mentioned (described as showing focal anaplasia and no foci of cartilage; no further details were available) [7]. Another case of a sporadic *DICER1*‑mutated botryoid fibroepithelial polyp of the parotid duct was published [9]. One of 30 sinonasal teratocarcinosarcomas had a *DICER1* mutation [10].

*DICER1* mutations predominantly represent germline variants but may also occur as sporadic (somatic) events [1]. For example, thyroblastoma, the most recently defined *DICER1*-altered entity, was found to represent acquired somatic disease in the majority of cases [11]. Individuals with the inherited *DICER1*-related tumor predisposition syndrome are at increased risk to develop the various tumors described in the introduction part. Accordingly, the patient’s medical and family history should be checked thoroughly for *DICER1*-suspicious neoplasms. In our case, the *DICER1* pathogenic variant showed a high allele frequency (VAF), but the site, age, and negative clinical history argued against a germline pathogenic variant, and this was confirmed molecularly. Our patient’s *DICER1* alteration is a somatic pathogenic variant probably with LOH of the wild type allele, hence the high VAF. Also, a germline p.Glu1813Gln has never been reported [12].

In summary, we herein describe the first case of a *DICER1*-mutated embryonal-like (botryoid-like) RMS of the sinonasal tract. Recognition of this rare presentation is important for clinical and genetic work-up of the affected patients. This case nicely highlights the value of standard morphology in predicting the genotype in specific entities. Although the current case is sporadic, it is significant to recognize these rare lesions as genetic testing for germline variants should be advised. Identification of more cases in the future may allow to address their possible association with the *DICER1*-related tumor predisposition syndrome more reliably.

## Data Availability

The datasets generated during and/or analyzed during the current study are not publicly available, but are available from the corresponding author on reasonable request.
